# Perilymph Fistula as a Complication of Eustachian Tube Dilation and Tympanoplasty

**DOI:** 10.1155/2022/5978757

**Published:** 2022-05-07

**Authors:** R. Kim, L. U. Scholtz, R. Jadeed, C. J. Pfeiffer, H. Sudhoff, I. Todt

**Affiliations:** Faculty of Medicine OWL, Bielefeld University, Campus Mitte, Department of Otolaryngology, Head and Neck Surgery, Bielefeld, Germany

## Abstract

Eustachian tube dilation (ETD) is an established, minimally invasive therapeutic approach for chronic eustachian tube dysfunction. The complications associated with performing a ETD are rare. A 22-year-old female patient presented with chronic otitis media on the right side and chronic obstructive tube dilation disorder on both sides. A type I tympanoplasty was performed on the right side because of a tympanic membrane perforation after a ETD on both sides without apparent complications. On the 5th postoperative day, she presented with headache, dizziness and hearing loss on the right side. There was a decrease of hearing threshold on the right side in the pure-tone audiogram and vHIT, cVEMP, and SVV were irregular. The *β*-2-transferrin test was positive. Since a right-sided perilymph fistula was suspected, an emergency tympanotomy was performed with a round window membrane cover with fascia on the right side. Intraoperatively, a regular, intact ossicular chain was found with a slightly moist middle ear mucosa. The round window membrane was covered by the promontorial lip. Under these measures, the patient's dizziness regressed. The right ear pure-tone threshold vHIT, cVEMP, and SVV normalized.

## 1. Introduction

Eustachian tube dysfunction causes clinical symptoms such as pressure sensation, tinnitus, deafness, hearing loss, and aural fullness. Furthermore, it can also cause chronic otitis media and cholesteatoma [1]. Eustachian tube dilation (ETD) is nowadays an established, minimally invasive therapeutic approach for chronic obstructive tubal disorder of the middle ear consisting of an insertion tool-based application of a balloon catheter with inflation up to a pressure of 10 mmHg over 2 minutes [2]. Several studies have already demonstrated the effectiveness of this procedure. The studies show that up to approximately 86% of patients benefit from ETD [2–4]. ETD is known to be a low-risk procedure [5]. So far, only rare complications such as hemorrhage, hearing loss, and air emphysema have been reported with 3%, 0.3%, and 0.27% occurrence rates, respectively [1, 6].

## 2. Case Presentation

**Figure 1:**
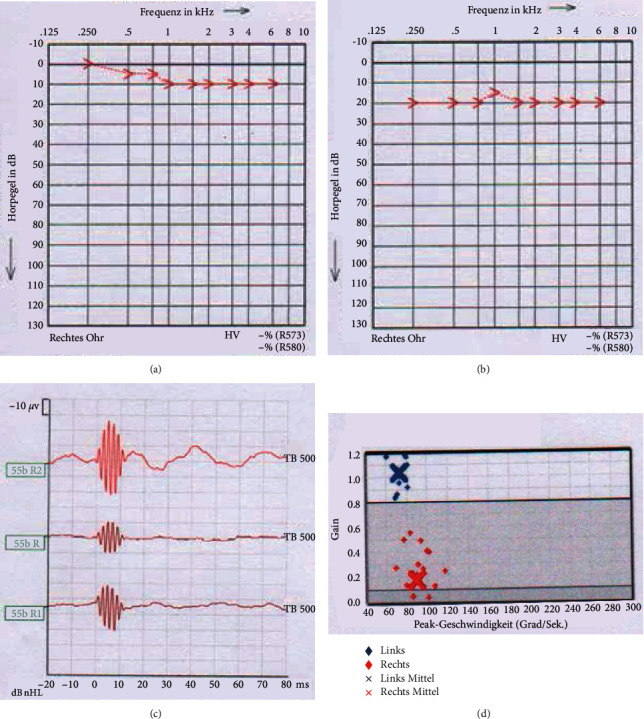
(a) PTA on the 1st postoperative day after tympanoplasty type I on the right and ETD on both sides. Regular BC threshold on the right. (b) PTA on day 5 postoperative tympanoplasty type I right and ETD bilateral. BC drop of 10–20 dB pantonal right. (c) No response when examining the cVEMP. (d) vHIT of the lateral semicircular canal. Right gain of 0.18, left 1.03.

A 22-year-old female patient presented with chronic otitis media on the right and chronic obstructive tubal disorder on both sides. A prior conservative therapy with a topical steroid spray and Valsava trials had been performed to treat the tubal dysfunction. The patient reported right-sided hearing loss and otalgia, a pressure sensation and no whistling on a Valsava trial. Clinically, there was an inflamed tympanic membrane with a small perforation on the right side. The Weber test lateralized to the right and a positive Rinne test on both sides was performed. Pure-tone audiometry (PTA) showed an almost symmetrical bone conductive threshold on both sides. A computed tomography (CT) scan of the middle ear showed no signs of a cholesteatoma. We performed a right tympanoplasty type I after a ETD on both sides. The postoperative PTA showed a regular bone conduction on the right side (Figure 1(a)). On the second postoperative day, the patient reported headache and fluid secretion from the nose. To control rhinoliquorrhoea, a neuropad was inserted endonasally. Since otologic symptoms were not present, a tympanotomy was not performed. A sample was sent for beta-2-transferrin analysis. However, the symptoms regressed spontaneously on the following day and the patient was discharged. At the time of discharge, the results of the *β*-2-transferrin test were still pending. Two days after discharge, on the 5th postoperative day, the patient came to our ENT emergency room with renewed cephalgia, dizziness, and hearing loss on the right side. A bone conduction (BC) drop of 10–20 dB on the right side was found in the PTA (Figure 1(b)). In the examination of the cervical vestibular evoked myogenic potentials (cVEMP), no response was detected (Figure 1(c)). The video head impulse test (vHIT) of the lateral semicircular canal was abnormal (Figure 1(d)). CT scanning showed no intra cochlea air and no bony dehiscence. The beta-2-transferrin analysis, which was taken on the second postoperative day, was positive. On suspicion of a perilymphatic fistula, a tympanotomy with a round window membrane coverage with fascia and fibrin glue on the right side was performed as an emergency measure.

Intraoperatively, there was no evidence of mechanical damage of the ossicular change (e.g., dislocation of the stapes) but a visually moistured mucosa of the middle ear. A rupture of the round window membrane was not visible because of a covering promontorial lip. Perioperatively, high doses of prednisolone and an antibiotic were given intravenously.

**Figure 2:**
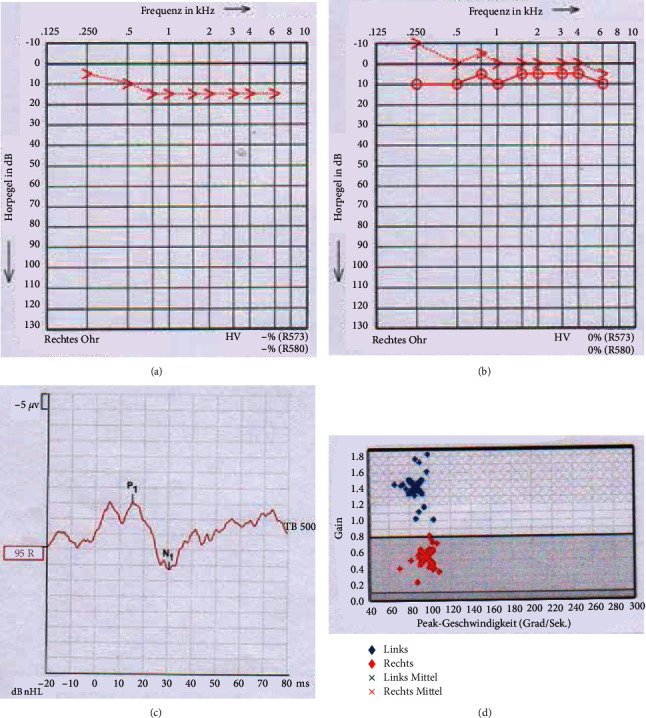
(a) PTA on the 1st postoperative day after tympanoscopy with round window coverage on the right. Increase of the BC threshold of 5–15 dB pantonal. (b) PTA 3 weeks after tympanoscopy with round window coverage on the right. Complete regression of the BC threshold on the right. (c) Presence of a response when examining the cVEMP. (d) vHIT of the right lateral semicircular canal. Increase of gain up to 0.58.

Under these measures, the symptoms regressed rapidly. On the 1st postoperative day, the PTA showed an increase of the bone conduction (BC) threshold of 5–15 dB pantonal (Figure 2(a)) and a subjective significant improvement of the dizziness symptoms. We performed a beta-2-transferrin analysis endonasally again after 3 weeks, this time being negative. Even three weeks after the tympanoscopy with round window coverage on the right side, the BC threshold had completely regressed in the PTA (Figure 2(b)), a response in cVEMP was detected (Figure 2(c)) and a clear increase of the gain up to 0.58 of the lateral semicircular canal (SCC) on the right side was shown in the vHIT (Figure 2(d)). The SVV normalized from 4.8° to 2.4°. The patient was symptom-free at this time.

## 3. Discussion

ETD is a low-risk, minimally invasive treatment option for chronic obstructive tubal dysfunction, which is nowadays used with a high therapeutic efficacy [7]. Rare risks reported include bleeding, hearing loss, and air emphysema with 3%, 0.3%, and 0.27% occurrence rate, respectively [1, 6]. In our case report, we presented a patient with a perilymphatic fistula after ETD and tympanoplasty who achieved rapid symptom relief with prompt round window coverage. We assume that the patient's perilymph fistula was caused by pressure change of the middle ear. Even a tiny leak at the lower edge by a mechanical cause [8] related to the tympanoplasty cannot be completely ruled out, but seems to us unlikely, since the tympanoplasty procedure was quite limited including positioning a small piece of underlay cartilage without affection of the ossicular chain. The possibility of a round window rupture has already been mentioned as a mechanism of hearing loss after ETD [1]. In animal experiments, it was shown that the pressure change of the middle ear is influenced by maximum inflation pressure as well as speed of balloon inflation during ETD, but the pressure changes of the middle ear are too low for a barotrauma of the ear structures [9]. The individual anatomy of the patient as well as the extent of pneumatisation of the middle ear, the mastoid as well as the nature of the round window under chronic middle ear inflammation could therefore underlie a present barotrauma. The *β*-2-transferrin test is nowadays the clinically common method for the detection of a cerebrospinal fluid (CSF) fistula. There are limitations with regard to the detection of a perilymph fistula [10]. Studies show that the *β*-2-transferrin test has a low sensitivity of approx. 29–66.7% and a high specificity of approx. 100% in the detection of a perilymph fistula [11, 12]. In our case, the endonasal *β*-2-transferrin test was positive after performing a type I tympanoplasty and ETD. Accordingly, a tympanotomy was performed with a round window membrane cover with fascia on the right side. There was no intraoperative evidence of mechanical damage (e.g., stapes dislocation). The constellation of hearing loss with dizziness and pathological findings with a positive *β*-2-transferrin test as well as the absence of clinical symptoms after surgical coverage and a negative *β*-2-transferrin test suggest the presence of an initial perilymphatic fistula after ETD. Since the ossicles were not intervened, but only a Typ I with a small cartilage underlay was performed, a tympanoplasty-related PLF seems unlikely. Recently, an ELISA-based diagnostical method for perilymph fistula is regarded to be promising. This method detects a perilymph-specific protein, cochlin-tomoprotein (CTP), as a diagnostic marker. The sensitivity and specificity of the test accounts for 86.4% and 100%, respectively [13]. This new clinical test might help with recognizing perilymph fistula and therewith ensure treatment.

Scarring in chamber formation is often observed in cases of chronic otitis media. Since the tube dilatation was performed before the tympanoplasty, we assume that there was a pressure or mechanical transfer of force to the round window due to scarring and chamber formation.

## 4. Conclusion

In this case report, we present a patient with a perilymphatic fistula after tubal dilation, which is most likely caused by a pressure change of the middle ear as a result of the ETD. After a prompt tympanoscopy with round window coverage on the right side, a rapid symptom improvement with complete regression of PTA threshold and vertigo symptoms and vestibular resceptor function could be achieved. In retrospect, it would have been preferable to have removed the bony lip of the round window (RW) niche to directly visualize the RW membrane. To avoid hearing loss after ETD, continued attention should be paid to slow catheter insertion and removal [1]. In addition, if fistula symptoms occur after ETD, rapid diagnosis and prompt therapeutic intervention would be essential to eliminate the complication.

## Data Availability

The data are available from the corresponding author upon request.
